# Antiseizure medications for primary and secondary seizure prevention after stroke

**DOI:** 10.3389/fneur.2025.1648064

**Published:** 2025-07-08

**Authors:** Zoe C. Wolcott, Brin E. Freund, William O. Tatum, Anteneh M. Feyissa

**Affiliations:** Department of Neurology, Mayo Clinic Florida, Jacksonville, FL, United States

**Keywords:** antiseizure medication, early seizure, late seizure, post-stroke seizures, stroke-related epilepsy, stroke, symptomatic epilepsy

## Abstract

Post-stroke seizures (PSS) and post-stroke epilepsy (PSE) are serious complications of cerebrovascular disease, contributing to morbidity, delayed recovery, cognitive decline, and mortality. PSS are classified as early (within 7 days) or late (after 7 days), with late-onset seizures often signaling the development of PSE. As stroke survival improves, the incidence of PSS continues to rise. Risk factors include cortical involvement, large or severe strokes, and early seizures. Although antiseizure medications (ASMs) are central to management, their use is limited by a lack of high-quality trials and reliable predictive tools. Routine primary prophylaxis is generally discouraged, except in high-risk patients—such as those with hemorrhagic stroke or severe cortical damage—while secondary prophylaxis after unprovoked seizures remains standard. Evidence supporting specific ASMs is limited, but lamotrigine and levetiracetam are considered reasonable first-line options. ASM selection should be individualized, particularly in older adults or those with cardiovascular or cognitive comorbidities, for whom older, enzyme-inducing ASMs carry greater risks. Withdrawal is often recommended after early seizures, but managing established PSE remains challenging without validated biomarkers. High-quality trials are urgently needed to evaluate the efficacy, safety, and tolerability of ASMs in post-stroke seizure prevention. Advancing the field also requires robust validation studies, improved prediction models, and personalized treatment strategies. This minireview summarizes current approaches to ASM use in PSS, with an emphasis on clinical decision-making for initiation and discontinuation.

## 1 Introduction

Post-stroke seizures (PSS) are classified as early (within 7 days) or late (after 7 days). Early seizures, or acute symptomatic seizures (ASS), result from transient neurochemical changes post-stroke and are not typically epileptic. Late seizures, or unprovoked seizures, stem from lasting structural brain changes and signify post-stroke epilepsy (PSE). The 7-day cutoff is widely accepted and aligns with underlying pathophysiology (1). Early seizures occur in 3–6% of stroke patients, more commonly in hemorrhagic (10–16%) than ischemic strokes (2–4%) (2, 3). Stroke causes 73% of acute symptomatic seizures in adults. Late seizures affect 3–5% using the 7-day definition, with incidence up to 12%. According to the International League Against Epilepsy (ILAE), PSE can be diagnosed after a single unprovoked seizure, as it reflects an enduring brain change with a high recurrence risk (>60% over 10 years) (1). Redefining PSE to include single late seizures has raised incidence estimates to 8–12% (1).

Risk factors for PSS include cortical involvement, severe or large strokes, and early seizures (2, 4, 5). Hemorrhagic strokes carry a higher PSE risk (12.4%) than ischemic ones (6.4%). Additional predictors include ICH volume, younger age, hyponatremia, alcohol use, and premorbid disability (4). Stroke treatments, including decompressive craniectomy, craniotomy, intravenous alteplase, or endovascular treatment, are also considered risk factors (3). Routine scalp electrocephalograpm (EEG) has not reliably predicted PSE, but focal epileptiform discharges and lateralized periodic patterns may carry prognostic value (6). Prediction models like the SeLECT score exist but need further validation before widespread use (7).

Studies indicate that PSS is associated with worse functional outcomes and increased disability. Patients with PSS have significantly higher modified Rankin Scale scores and greater odds of poor outcome (3). PSE also contributes to long-term morbidity. There is growing evidence linking PSE with increased dementia risk. A 2.5-fold higher dementia incidence was reported in young stroke survivors with seizures, and pooled analyses confirm that PSS is independently associated with dementia (8). This suggests a feed-forward relationship among stroke, PSS, and neurodegeneration (8).

This minireview discusses antiseizure medication (ASM) therapies for managing PSS, including clinical considerations for initiating and discontinuing treatment.

## 2 Primary prophylaxis

Routine primary prophylaxis ASMs after stroke is generally not recommended due to the low incidence of PSS or PSE and the significant risk of adverse drug reactions (ADRs), especially in older adults with comorbidities (9, 10). Professional guidelines reflect this: the European Stroke Organization gives a weak recommendation against primary prophylaxis due to very low-quality evidence, and the American Heart Association/American Stroke Association similarly advises against routine ASM use, noting that potential harms outweigh benefits for most survivors (10).

For most patients, harms outweigh the benefits of preventing a first seizure. However, certain high-risk groups may warrant selective primary prophylaxis briefly. Even then, decisions must carefully weigh seizure risk against ASM tolerability and ADRs (11, 12). Tools such as the SeLECT score for ischemic stroke and the CAVE and 2HELPS2B scores for ICH help quantify seizure risk (4, 12). These models include factors like cortical involvement, NIHSS severity, early seizure, and MCA distribution infarcts. Despite this, primary prophylaxis is rarely recommended, even in high-risk patients, as efficacy evidence remains sparse and low quality (9, 11).

Evidence for primary ASM prophylaxis after hemorrhagic stroke is limited. Two randomized trials assessed this: one comparing valproate to placebo in 72 ICH patients showed no significant benefit (13), while the PEACH trial testing levetiracetam yielded mixed results—some reduction in electrographic seizures but no effect on clinical seizures (14). Both were underpowered, with the PEACH trial halted early due to poor recruitment. A Cochrane review incorporating these studies concluded ASMs do not effectively prevent post-stroke seizures, rating the evidence as low quality due to imprecision (9). No trials support long-term prophylaxis for late unprovoked seizures. Two small studies on short-term prophylaxis post-ICH were inconclusive (11, 12).

Observational studies provide important insights. A real-world study in older adults with acute ischemic stroke found higher 30-day mortality among those receiving seizure prophylaxis within 7 days, raising concerns about net benefit (15). Decision models favor secondary over primary prophylaxis. One model showed that starting ASMs after a seizure consistently yields better quality-adjusted life years (QALYs) than primary prophylaxis (11). Another model for ICH suggested short-term prophylaxis (e.g., 7 days) may benefit select high-risk patients, but long-term use generally leads to worse outcomes due to ADRs (12, 16).

In conclusion, current evidence does not support routine primary prophylaxis with ASMs, though select high-risk patients may be considered. When ASMs are indicated, lamotrigine, levetiracetam, lacosamide, and oxcarbazepine are preferred for their safety profiles (15, 17).

## 3 Secondary prophylaxis

Secondary prophylaxis refers to the initiation of ASMs following a seizure in a stroke patient to prevent recurrence. Patients with early seizures carry a relatively low risk of immediate recurrence (10–20%) and a moderate risk of late recurrence (~30% over 10 years) (2, 3). Consequently, long-term ASM therapy is typically not recommended after a single early seizure. However, in cases of acute symptomatic status epilepticus, extended treatment is warranted (14). Short-term ASM use during the acute phase may also be considered to reduce excitotoxicity, with subsequent tapering.

In contrast, patients who experience late seizures are at a substantially higher risk of recurrence—exceeding 70% within 10 years. According to the ILAE, a single unprovoked seizure in this setting qualifies for a diagnosis of epilepsy (1). Discontinuing ASMs in these patients results in relapse rates over 50%, underscoring the need for long-term therapy (18). Therefore, secondary prophylaxis is generally recommended for all patients with post-stroke unprovoked seizures (19). Table 1 summarizes the commonly used ASMs for secondary prophylaxis, along with key clinical considerations to guide individualized treatment decisions based on current evidence.

**Table 1:** ASM options for primary and secondary prophylaxis of post-stroke seizures.

Category	ASMs	Key considerations
First-line	Levetiracetam/Brivaracetam	Favorable safety and tolerability Limited drug interactions Potential antiepileptogenic effects Behavioral side effects (e.g., irritability, depression)
	Lamotrigine	Requires slow titration to reduce risk of rash (SJS) Low discontinuation rates Minimal drug interactions ECG screening recommended in older adults or those with cardiac disease
	Eslicarbazepine	Enhances slow sodium channel inactivation Risk of hyponatremia, especially in elderly Minimal hepatic metabolism Cost may be a limiting factor
	Lacosamide	Well tolerated Minimal drug interactions Avoid in patients with AV block May benefit nonconvulsive status epilepticus in elderly with stroke
Second-line	Gabapentin/Pregabalin	Useful for neuropathy, central stroke pain, and anxiety Risk of dizziness and sedation, especially in elderly
	Oxcarbazepine	Risk of dose-dependent hyponatremia Possible interaction with DOACs Lower enzyme induction than carbamazepine
	Clobazam	Adjunctive option Use lowest effective dose to minimize sedation and fall risk
	Perampanel	Adjunctive therapy Monitor for behavioral effects (e.g., aggression, irritability, depression)
Consider avoiding	Phenytoin	Strong enzyme inducer; many drug–drug interactions (e.g., anticoagulants, statins, antihypertensives) May impair stroke recovery Associated with increased vascular risk Narrow therapeutic window; high discontinuation rate
	Carbamazepine	Enzyme inducer with extensive drug interactions Increased mortality risk in elderly stroke patients
	Valproic Acid	Enzyme inhibitor Risk of coagulation abnormalities May cause weight gain and hyperammonemia Not preferred in elderly
	Zonisamide/Topiramate	Cognitive side effects (avoid in patients with cognitive impairment) Topiramate may aid in weight loss or relief of migraine/stroke-related pain

ASMs, Antiseizure medications; AV, Atrioventricular; DOACs, Direct oral anticoagulats; ECG, Electrocardiogram; SJS, Stevens-Johnson Syndrome.

### 3.1 Efficacy considerations

Identifying the most effective ASM for PSE is challenging due to this population’s lack of high-quality randomized controlled trials. European guidelines have not found any ASM or class with clear superiority for PSE (10). Thus, current recommendations rely mainly on expert consensus and data from studies of older adults with diverse epilepsy causes (11, 14, 17). Recent network meta-analyses frequently highlight levetiracetam and lamotrigine as preferred ASMs (17). Also, ASMs acting via slow sodium channel inactivation, such as lacosamide and eslicarbazepine, show promise in observational studies and meta-analyses, with low seizure recurrence rates. Conversely, enzyme-inducing ASMs (EI-ASMs) carbamazepine and phenytoin raise issues due to interactions with anticoagulants (11, 15). Phenytoin’s narrow therapeutic window and poor tolerability further limit its use (11, 15).

Levetiracetam may have antiepileptogenic effects and has demonstrated efficacy in randomized trials (15, 17). Observational data suggest better functional outcomes with levetiracetam compared to phenytoin (15). In one study, lamotrigine had a low adverse event profile and was linked to lower mortality than carbamazepine, although cardiac monitoring is recommended in older adults (14). Lacosamide and eslicarbazepine, which modulate sodium channels via slow inactivation, show promise in seizure control and are well tolerated (15, 17). However, hyponatremia is a potential side effect, particularly with eslicarbazepine and oxcarbazepine in elderly patients (15, 17). Lacosamide has also shown efficacy in treating non-convulsive status epilepticus in elderly stroke patients (17). Experts also favor Gabapentin as a second-generation option (17).

### 3.2 Adverse effects and drug interactions considerations

Anti-seizure medications can cause a range of ADRs and drug interactions, especially important in stroke patients with comorbidities and polypharmacy. These side effects may impede neurological recovery, hinder rehabilitation, and increase morbidity (11, 14, 15, 19). Common issues include sedation, dizziness, and tremor. Topiramate is linked to cognitive impairment, while newer agents like levetiracetam and perampanel can cause behavioral disturbances such as anxiety, irritability, and depression (11, 14, 17). Levetiracetam is also associated with somnolence and fatigue, which may raise fall risk (17). Phenytoin has well-known adverse effects, including ataxia, arrhythmias, and hypersensitivity reactions, and is tied to poorer recovery (11, 14).

The impact of ASMs on post-stroke outcomes remains uncertain. While seizures are linked to poorer recovery, it is unclear if ASM treatment independently affects outcomes. Observational studies suggest phenytoin is associated with worse recovery compared to levetiracetam (15). GABAergic agents like benzodiazepines and phenobarbital, and older ASMs such as phenytoin, may also impair motor and cognitive recovery and should be used cautiously (11, 19). High discontinuation rates due to side effects or inefficacy can also compromise seizure control. Among ASMs, lamotrigine shows the lowest discontinuation rates, though evidence quality is low (15, 17). Vertigo and fatigue are common across all ASM classes.

Drug interactions are a major concern in stroke patients, especially given frequent use of anticoagulants, statins, and antihypertensives. EI-ASMs such as carbamazepine, phenytoin, phenobarbital, and primidone can alter the metabolism of critical medications, potentially lowering serum levels of direct oral anticoagulants (DOACs) and statins, increasing thrombotic and lipid-related risks (11, 14, 17). Although levetiracetam has been suspected to affect DOACs via P-glycoprotein modulation in animals, clinical data do not support this (14). EI-ASMs are also linked to adverse metabolic effects, including increased homocysteine, uric acid, and inflammatory markers (14). Newer ASMs like lamotrigine, levetiracetam, lacosamide, and eslicarbazepine have more favorable interaction profiles, higher tolerability, and minimal drug impact, making them preferred choices (11, 14, 17).

### 3.3 Comorbidity considerations

When selecting an ASM, underlying comorbidities must be considered, as they significantly affect tolerability and safety. Older adults, who comprise most stroke survivors, are especially vulnerable to side effects. Age-related changes in pharmacokinetics, frailty, and polypharmacy increase the risk of adverse outcomes (11, 14, 17, 19). For instance, gabapentin, eslicarbazepine, and oxcarbazepine may cause dizziness or hyponatremia (11, 17). Cardiovascular comorbidity is also common post-stroke. ASMs such as phenytoin can induce arrhythmias and are linked to higher mortality. Lamotrigine, although generally safe, carries a U.S. FDA warning for potential cardiac effects and should be preceded by ECG screening in patients with known cardiac disease or those over 60 (14).

Medication interactions are particularly important in patients taking anticoagulants or statins. EI-ASMs reduce the serum concentration of many drugs, including DOACs, and may compromise secondary stroke prevention (11, 14). Clinical evidence does not support an interaction between levetiracetam and DOACs, although caution remains (14). EI-ASMs may also reduce statin efficacy and adversely affect lipid metabolism and vascular inflammation (11, 14).

Cognitive and psychiatric comorbidities are also important considerations. ASMs like topiramate may exacerbate cognitive dysfunction, while levetiracetam and perampanel can cause mood disturbances (11, 17). These effects are especially relevant in stroke patients with pre-existing or stroke-induced cognitive or psychiatric disorders. Sedating side effects such as somnolence, dizziness, or ataxia elevate fall risk, which can be catastrophic in patients with impaired mobility or osteoporosis (11, 14). Adherence is another concern, with many older ASMs having high discontinuation rates due to poor tolerability. Lamotrigine is associated with fewer ADRs and drug discontinuations, whereas phenytoin and carbamazepine are poorly tolerated (11, 14, 17). ASM choice must be tailored based on comorbidities, concurrent medications, and fall or cognitive risk (19).

### 3.4 Stroke recurrence considerations

Older EI-ASMs can reduce serum levels of anticoagulants and statins, potentially increasing vascular risk by elevating cholesterol and inflammatory biomarkers (11, 14). Phenytoin has been associated with higher mortality in PSS and may negatively impact motor recovery (11, 14, 15). Although valproate was linked to improved outcomes in one intracerebral hemorrhage trial, it is generally considered less favorable due to coagulation concerns and metabolic side effects, including weight gain and increased vascular risk (13). Phenobarbital and benzodiazepines can impair neurological recovery and have been correlated with increased mortality (11, 14).

Age-accelerated atherosclerosis is well-documented in patients with epilepsy. Long-term treatment with ASMs may contribute to vascular endothelial dysfunction and elevate the risk of developing atherosclerosis (20, 21). Studies have reported that patients taking carbamazepine, phenytoin, or valproic acid exhibit increased arterial stiffness and greater intima–media thickness in the cervical carotid arteries, which correlate with the duration of ASM therapy (20, 21).

Some non-ASM therapies may provide dual benefits. Statins, while not ASMs, have been associated with reduced risks of both acute symptomatic seizures and PSE, especially at higher doses and longer durations (11). Their neuroprotective effects are thought to arise from anti-inflammatory properties, reduced excitotoxicity, and enhanced blood–brain barrier stability (6, 14). In animal models, statins may also potentiate ASM efficacy (6). Clinically, statin use correlates with lower seizure recurrence and fewer epilepsy-related hospitalizations in patients with cardiovascular disease (14). Certain antihypertensive agents and diuretics may have ancillary antiepileptic properties. Angiotensin receptor blockers (ARBs) like losartan and telmisartan might reduce epileptogenesis mediated by blood–brain barrier disruption through TGF-*β* inhibition (6). Diuretics like thiazides and furosemide have shown seizure-reducing effects in both animal models and clinical settings (6).

## 4 Duration of therapy

Current evidence and expert consensus generally advise against routine long-term ASM initiation for primary prophylaxis in patients without post-stroke seizures (9–11, 17). Decision analyses consistently show that starting ASM only after a seizure—secondary prophylaxis—results in better outcomes, measured by quality-adjusted life years (QALYs), compared to primary prophylaxis (9, 11, 16). For example, one comprehensive decision analysis found that long-term primary prophylaxis yielded the lowest QALYs, supporting recommendations against prophylactic ASM use immediately after acute ischemic stroke (11, 16). Clinical trials have not demonstrated that short-term ASM use after stroke prevents epilepsy; ASMs function as antiseizure agents rather than antiepileptogenic therapies (9, 14).

When ASMs are initiated acutely—typically for early seizures within the first 7 days post-stroke—they should be prescribed for a limited duration. Clinical guidelines recommend early withdrawal of ASMs after the acute phase, generally within 1 to 2 weeks (9, 11, 17). In spontaneous ICH (sICH), decision analyses highlight the advantage of short-term (7-day) ASM treatment, urging clinicians to document discontinuation plans in prescriptions, discharge summaries, and patient education (11, 16). Although specific tapering protocols are not well established, consensus stresses early withdrawal due to low recurrence risk and no demonstrated benefit from prolonged primary prophylaxis (9, 11, 17).

Withdrawal of ASMs following secondary prophylaxis depends on whether treatment was initiated for an early or a late seizure consistent with PSE. For early seizures, guidelines recommend limiting ASM therapy to the acute phase—typically 1 to 2 weeks, or 7 days for sICH (9, 17). This is supported by the relatively low seizure recurrence risk (10–20%) and a moderate 10-year risk (~30%) of developing late unprovoked seizures (1, 3). Prolonged treatment after an early seizure does not improve outcomes and may increase adverse effects. Thus, routine ASM discontinuation is advised (9, 17).

In contrast, ASM withdrawal in patients with PSE is more complex and must be individualized. These patients carry a high risk of recurrence after withdrawal—over 50% in some studies—due to the symptomatic, lesional nature of their epilepsy (1, 20). The highest relapse risk occurs within the first 12 months post-withdrawal but may persist for years. Factors influencing withdrawal include age at onset and withdrawal, epilepsy etiology, seizure type, EEG findings, remission duration, and overall burden (11, 17, 22). Warning signs against withdrawal include focal seizures, short seizure-free intervals, abnormal neurological exams, and epileptiform EEG activity. In elderly patients, withdrawal is further complicated by seizure risks and drug interactions, even though relapse rates may be lower in late-onset epilepsy (17, 22). While rationale for withdrawal or continuation is clear, specific tapering protocols remain poorly defined (9, 11, 17).

### 4.1 Biomarkers to guide therapy duration

EEG and neuroimaging biomarkers have been extensively studied for their roles in predicting PSE, yet their utility in guiding ASM withdrawal remains limited and largely indirect (6, 20). EEG abnormalities—such as background asymmetry, interictal spikes, sharp waves, and periodic discharges—are associated with increased PSE risk (6). Early EEG findings, particularly ictal activity, can predict epilepsy development; however, their role in ASM discontinuation decisions is not well established or validated (6). Persistent EEG abnormalities may serve as cautionary indicators, but stopping ASM should primarily rely on clinical judgment, individualized risk assessment, seizure history, and patient-specific factors rather than validated biomarker thresholds (6, 20).

Similarly, neuroimaging biomarkers show promise in forecasting PSE risk. Features like cortical involvement, lesion size, and cortical superficial siderosis correlate with epileptogenesis (6). Advanced imaging techniques assessing blood–brain barrier permeability or glutamate concentration are under research (6, 18). Despite these advances, existing evidence does not support routine use of standard or advanced neuroimaging as reliable biomarkers for guiding ASM withdrawal (6, 22). While imaging findings may influence the initial decision to start ASM therapy, they do not reliably predict the timing or safety of discontinuation (6, 22).

## 5 Non-pharmacologic treatments

While ASMs remain the cornerstone of PSS management, non-pharmacologic options also play a key role for patients with drug-resistant epilepsy (DRE). These include resective epilepsy surgery and neuromodulation therapies such as vagus nerve stimulation (VNS) and responsive neurostimulation (RNS) (11, 14, 17, 19).

Surgery is a viable option for select patients with DRE whose seizures arise from a well-localized epileptogenic zone. Surgical candidacy and evaluation follow principles similar to those in other focal epilepsies. Pre-surgical assessment typically involves multimodal imaging—high-resolution 3 T MRI, ictal SPECT, PET—and intracranial EEG monitoring to localize seizure onset (11, 17). In properly selected patients, resective surgery can significantly reduce seizure burden or achieve remission (17).

VNS has become an important adjunctive treatment for DRE. Beyond seizure control, VNS shows promise as a targeted plasticity intervention post-ischemic stroke by modulating brain nuclei linked to neural recovery. Animal studies show that combining VNS with rehabilitative training enhances motor recovery beyond rehabilitation alone (14, 19). Significantly VNS, paired with high-dose occupational therapy, has been shown to be effective in improving upper limb function among patients with ischemic stroke and received regulatory approval from FDA (23, 24). RNS, which delivers electrical stimulation in response to abnormal brain activity, is another option for refractory cases (14, 19). These interventions are generally reserved for patients who have failed adequate ASM trials and meet criteria for DRE, offering hope for improved seizure control and functional outcomes (11, 14, 17, 19).

## 6 Future directions

Advancements in managing post-stroke seizures and epilepsy depend on several key priorities. High-quality randomized controlled trials are urgently needed to assess the efficacy, safety, and tolerability of ASMs for both primary and secondary seizure prevention following ischemic stroke (9, 11, 14, 17). These trials should be double-blind, placebo-controlled, adequately powered, and focused on clinically meaningful outcomes such as seizure freedom and ASM withdrawal rates (9, 11, 17).

Improved risk stratification through validated clinical prediction models, integrated with molecular, imaging, and electrophysiologic biomarkers, is essential (6, 22). Emerging artificial intelligence (AI) techniques offer promise in enhancing EEG and neuroimaging analyses, enabling more personalized assessments and targeted prophylactic strategies (6, 18).

Further research into the underlying pathophysiology—especially excitotoxicity and blood–brain barrier disruption—may identify novel therapeutic targets and biomarkers (6, 14, 18). Advanced imaging methods like GluCEST MRI and permeability imaging are central to these efforts (18). Additionally, exploring the anti-epileptogenic effects of existing drugs such as statins and novel agents like rapamycin could open new preventive avenues (6, 14).

**Figure 1:**
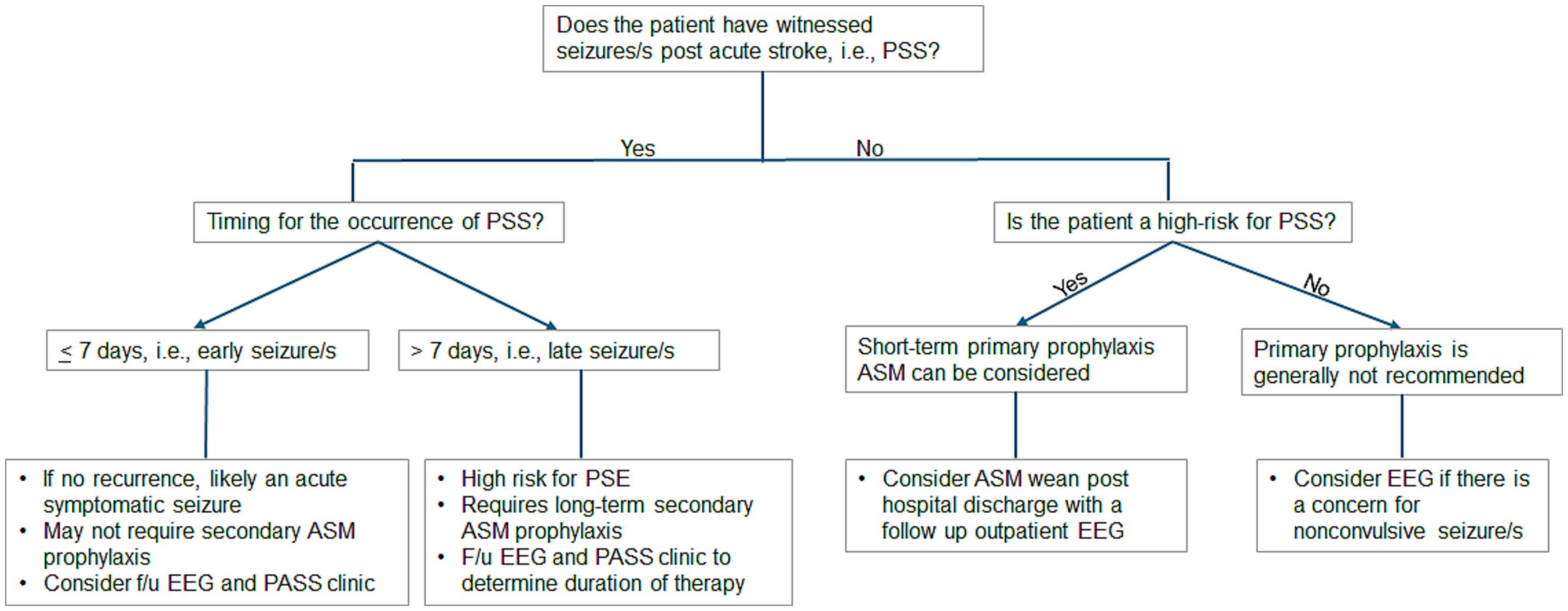
Proposed algorithm for decision-making regarding primary and secondary ASM prophylaxis in patients presenting with PSS. ASM, antiseizure medication; EEG, electroencephalogram; F/u, follow-up; PASS, Post-Acute Symptomatic Seizure; PSS, poststroke seizure.

Optimal treatment protocols—including timing, choice of ASM, dosing, duration, and withdrawal—remain incompletely defined and must be individualized based on factors such as comorbidities, medication tolerance, adherence, and risk of seizure recurrence (11, 14, 17). Figure 1 summarizes our proposed algorithm for decision-making regarding primary and secondary ASM prophylaxis in patients presenting with PSS.

Translating these advances into practice demands rigorously designed trials to validate experimental and observational findings. Future research must generate robust evidence, improve predictive accuracy, clarify mechanistic pathways, and personalize therapies to enhance outcomes for stroke survivors vulnerable to epilepsy (6, 11, 14, 17).

## 7 Conclusion

PSS and PSE are significant complications of cerebrovascular disease, contributing to morbidity, impaired recovery, cognitive decline, and increased mortality. Management remains limited by a lack of high-quality trials and validated predictive tools. Primary prophylaxis with ASMs is generally discouraged except in high-risk groups (e.g., hemorrhagic stroke, severe cortical injury, prior acute seizures), while secondary prophylaxis after unprovoked seizures is standard.

Newer ASMs like levetiracetam, lamotrigine, lacosamide, and eslicarbazepine are preferred for their safety and minimal interactions. Treatment should be individualized, especially in older patients or those with cardiovascular and cognitive comorbidities, where older EI-ASMs pose greater risks. ASM withdrawal is advised after early seizures, but decisions in established PSE remain complex due to limited biomarker guidance. Non-pharmacologic options, including surgery and neuromodulation, are valuable for drug-resistant cases and may support rehabilitation. Adjunctive therapies such as statins and certain antihypertensives show promise for seizure prevention and need further study.

Ultimately, improving outcomes in PSS and PSE requires well-designed trials, refined prediction models, and integration of emerging biomarkers. As understanding of vascular injury and epileptogenesis evolves, a more personalized, mechanism-driven approach to seizure prevention in stroke survivors is both necessary and achievable.
